# Associations Between Fitness, Physical Activity, and Fatness in Preschool Children With Typical and Atypical Motor Coordination

**DOI:** 10.3389/fped.2022.756862

**Published:** 2022-04-15

**Authors:** Shelley E. Keating, Gregore I. Mielke, Sara King-Dowling, Brian W. Timmons, Matthew Kwan, John Cairney

**Affiliations:** ^1^School of Human Movement and Nutrition Sciences, The University of Queensland, St Lucia, QLD, Australia; ^2^Infant and Child Health (INCH) Lab, Department of Family Medicine, McMaster University, Hamilton, ON, Canada; ^3^Division of Oncology, The Children’s Hospital of Philadelphia, Philadelphia, PA, United States; ^4^Department of Pediatrics, McMaster University, Hamilton, ON, Canada; ^5^Department of Child and Youth Studies, Brock University, St. Catharines, ON, Canada

**Keywords:** obesity, cardiorespiratory fitness, developmental coordination disorder, anaerobic capacity, aerobic capacity

## Abstract

**Purpose:**

Increased adiposity in children confers a higher risk of cardiovascular disease in later life, with low cardiorespiratory fitness strongly linked to poorer metabolic health. Children with motor coordination problems are likely to be less physically fit and at a higher risk of obesity. In this study, we examined the associations between aerobic and anaerobic fitness, device-measured physical activity, and body adiposity in children (aged 4–5 years) with typical and atypical motor coordination.

**Methods:**

Baseline data from the Coordination and Activity Tracking in CHildren (CATCH) cohort study were utilised. The assessments included aerobic and anaerobic fitness *via* time-to-exhaustion on Bruce treadmill test and normalised mean power on Wingate cycling test, respectively; light physical activity (LPA), moderate-to-vigorous physical activity (MVPA), and sedentary time *via* accelerometry; and body adiposity (%) *via* bioelectrical impedance analysis (BIA). The Movement Assessment Battery for Children-Second Edition (MABC-2) was used to assess motor coordination and classify children as typically developing (TD, >16th percentile) or at risk of developmental coordination disorder (DCD, ≤16th percentile). General linear regression models were fitted to examine associations.

**Results:**

The analyses included 495 participants (5.0 ± 0.6 years, 56% male, and body adiposity 22.7 ± 4.2%). Aerobic fitness (β = −0.006, *p* < 0.001) and MVPA (β = −0.018, *p* = 0.045) were negatively associated with body adiposity when adjusted for age, sex, and MABC-2 score. There was no relationship between sedentary time and body adiposity. There were no interactions of sex or MABC-2 score with any variable.

**Conclusion:**

Lower aerobic fitness and MVPA were associated with higher body adiposity in preschoolers, regardless of motor coordination. Interventions targetting improved aerobic fitness and MVPA are therefore warranted in both TD and atypically developing preschoolers. Whether maintaining high aerobic fitness in children with possible DCD confers protection against obesity requires longitudinal investigation.

## Introduction

An increased adiposity in childhood confers a higher risk of cardiovascular disease (CVD) and mortality in later life (1–3). Obesity has been shown to be the principle contributor to the cumulative risk of CVD (4). This is concerning as the global prevalence of childhood obesity was 7–8% in 2017 (5, 6), with 50–70% of children with obesity remaining obese in adulthood (7). Moreover, the metabolic milieu that precede the development of CVD (e.g., dyslipidaemia, elevated blood pressure, and hyperinsulinaemia) have been observed in adolescents and children as young as 9 years of age (8). Notably, low levels of cardiorespiratory fitness and an elevated body mass index (BMI, kg/m^2^) are strongly linked with these CVD risk factors (8). Beyond cardiometabolic health, obesity in childhood has also been linked with poorer academic skills and coping strategies (9).

There is a complex interplay between physical activity, aerobic fitness, and body adiposity. In both children and adolescents, higher cardiorespiratory fitness is associated with improved cardiovascular profiles in adulthood and a lower risk of premature death (1, 10). There is also an inverse association between moderate-to-vigorous physical activity (MVPA) and adiposity in children and adolescents (11). However, with only weak-to-moderate associations between physical activity and aerobic fitness in children (12–14), understanding the role of each component of health-related fitness and their relative influence on body adiposity is important to delineate to inform public health policy. In early-to-mid childhood (∼5–12 years, Tanner stage <3), associations between aerobic fitness and body adiposity have been observed (15–17), although conclusions have been limited by submaximal measures of aerobic fitness (15, 16) and the lack of control for device-measured physical activity levels and sedentary behaviour (15–17).

There is some evidence to suggest that obesity, determined by BMI, is associated with lower aerobic fitness in preschool-aged children (18, 19). However, there is a weak correlation between BMI and body adiposity (% fat mass) in the preschool-aged population (20), and aerobic fitness levels have been shown to differ by body adiposity irrespective of the BMI classification (16). Few studies in preschool-aged children have examined the relationships between the health-related components of physical fitness and body adiposity using accurate and reliable tools and adjusted for device-measured physical activity and sedentary time. Henriksson et al. (21) observed that a higher fat mass index (kg/m^2^ through air displacement plethysmography) was associated with a poorer cardiorespiratory and motor fitness (*via* 20 m shuttle run and 4 × 10 m shuttle run test, respectively), when adjusted for age, sex, and vigorous physical activity in 303 preschool-aged children (21). However, to our knowledge, no studies have examined these relationships within the context of motor coordination development using robust methods and controlling for habitual light physical activity (LPA), MVPA, and sedentary time.

Motor skills and motor function are likely determinants of physical activity participation and aerobic fitness in children and adolescents (22). Consequently, children with neurodevelopmental problems [for example, those with developmental coordination disorder (DCD)] have been shown to be less active, less physically fit, and are at a higher risk of having overweight or obesity than typically developing (TD) children (23). DCD affects 5–6% of the paediatric population and is characterised by impairments in fine and/or gross motor coordination impacting on everyday functions, such as play and academic ability (24). This is likely to impact on engagement in skill-related physical activity as children with DCD age and is a likely contributor to a decline in physical fitness. However, recent findings suggest that young children (aged 4–5 years) at risk for DCD are not yet deficient in physical activity accumulation (25), likely reflecting the unstructured patterns of play in this age group. Moreover, while there were no differences in body composition, there were differences in aerobic fitness in those with motor delays (26); however, the interactions between fitness, physical activity, sedentary time, and body composition are still unknown.

Describing the relationships and interactions between physical fitness, physical activity, sedentary time, and body adiposity in young childhood may help to understand predictors of body adiposity and inform targets for public health messages and lifestyle intervention for children to prevent obesity-related chronic diseases. Therefore, the primary aim of this study was to examine the associations between aerobic and anaerobic fitness, device-measured physical activity and sedentary time, and body adiposity in a cohort of young Canadian children (aged 4–5 years). We also aimed to examine the differences in associations between children with typical and atypical motor coordination. Based on the observations from BMI-derived associations and evidence in older children, we hypothesised that there would be an inverse relationship between aerobic and anaerobic fitness, MVPA, and body adiposity, and a positive association between sedentary time and body adiposity. With a paucity of evidence for the association between LPA and overall health outcomes, we hypothesised that there would be no associations between LPA and body adiposity. Finally, given the lack of differentiation in physical activity levels based on neurodevelopment in preschool-aged children, we further hypothesised that these relationships would be similar regardless of motor coordination classification in this population.

## Materials and Methods

### Participants

This study used cross-sectional baseline data from the Canadian longitudinal cohort study: Coordination and Activity Tracking in CHildren (CATCH). The CATCH study is a longitudinal cohort study including 588 children aged 4–5 years (48–71 months) at baseline (2015). Based on the child’s performance on a standardised motor test [Movement Assessment Battery for Children-Second Edition (MABC-2)], 301 children were classified as TD (overall MABC-2 score >16th percentile) and 287 were classified at risk for developmental coordination disorder (rDCD, overall MABC-2 score ≤16th percentile). The participants were recruited from a community-based sample from Hamilton, Ontario and the surrounding areas through community organisations and events, school mail outs, recruitment posters, and social media between October 2013 and June 2017. A detailed description of the design, eligibility, and outcome measures of the CATCH longitudinal cohort study (27) and the complete cohort baseline profile (28) have been previously published. In brief, children who were not eligible were those who did not speak/understand English, had low birthweight (<1500 g), or were diagnosed with a physical disability or medical condition that affects motor coordination (e.g., cerebral palsy or muscular dystrophy). Children were also ineligible if scoring an IQ < 70 based on the Kaufman Brief Intelligence Test, Second Edition (29), to exclude deficiencies that may be due to an intellectual delay. The legal guardians of participants signed a written informed consent before participating in data collection. The study was approved by the Hamilton Integrated Research Ethics Board. In this cross-sectional sub-study, children were also excluded if they did not have a valid measure of body fatness (*n* = 5), or valid measures of aerobic fitness (*n* = 4) or anaerobic fitness (*n* = 17) or device-measured physical activity (*n* = 75). Collectively, 93 children were excluded from the sub-study due to one or more missing variables. Further details on these missing data have been previously reported (25, 26).

The baseline study appointment included a motor coordination assessment, anthropometric measures, followed by non-invasive physical fitness measures including aerobic fitness, followed by anaerobic fitness after a minimum 10-min rest. The children were fitted with an accelerometer and the parents were instructed on its use. All questionnaires were completed by parents/guardians during the baseline visit.

### Measurements

#### Anthropometry and Body Composition

Height (cm) and the body mass (kg) were measured in duplicate without shoes and in light clothing using a stadiometer (SECA 264, Chino, CA, United States) and digital scale (SECA 896), respectively. A third measure was taken if repeat measures were >0.1 cm and >0.1 kg apart, respectively, with the average of the two closest measured used. BMI (kg/m^2^) was calculated and BMI percentile determined based on the growth charts of the US Centre for Disease Control and Prevention (30). Waist circumference was measured in duplicate midway between the top of the iliac crest and lowest rib against the skin during normal exhalation. A third measure was taken if the differences between the measures were >0.5 cm with the average of the two closest measures taken.

Body adiposity (body fat %) was assessed using bioelectrical impedance analysis (BIA) (RJL Systems – Quantum IV Body Composition Analyzer) *via* a whole-body impedance method using a tetrapolar model, with the child lying in the supine position. Fat-free mass was calculated using the standardised age-specific equations using the resistance values (31), validated against doubly labelled water, and percentage body fat was calculated as [(body weight − FFM)/body weight] × 100.

#### Aerobic Fitness

Aerobic fitness was assessed using the full Bruce protocol (32) on a treadmill (Valiant, Lode BV, Groningen, Netherlands). The full Bruce protocol has been shown to be a more accurate measure of maximal exercise fitness/endurance (i.e., time-to-exhaustion) in 4- and 5-year olds than the modified Bruce protocol. Participants began the test at a slow walking pace of 2.7 km/h at 10% grade for 3 min, with the speed and incline of the treadmill increasing every 3 min in a standardised manner until volitional fatigue. A researcher was positioned behind the child to ensure safety. The test was terminated when participants were unable to continue with increasing speed or grade, or refused to continue despite verbal encouragement. To assist with coordination and balance, the participants were instructed to hold onto the handrails of the treadmill for the duration of the test. The heart rate was continuously monitored *via* a chest-worn monitor (Polar H7, Polar Electro FTI, Kempele, Finland). Aerobic fitness was determined as the total time on the test. Maximal exertion was verified by the peak heart rate achieved and only the children who reached a maximum heart rate of 180 bpm or greater were included in analyses (33).

#### Anaerobic Fitness

Anaerobic fitness (muscle power) was assessed *via* the Wingate anaerobic cycling test using a paediatric cycle ergometer (Paediatric Corival, LODE). The maximum pedal speed was firstly determined by the participants sprinting as fast as possible against the initial resistance of the ergometer for 20–30 s. Then the participants were asked to stop pedalling and once the revolutions per minute (rpm) dropped below 50% of the maximum pedalling speed, the test was commenced. When the pedal speed reached 80% of the maximum pedal speed, a predetermined breaking force of 0.55 N/m/kg was applied. A verbal encouragement was provided to the participants to keep pedalling as fast as they could for 30 s. The power outputs were determined using the Wingate software package (Lode BV). The mean power was calculated as the average power over 30 s and normalised to body mass (W/kg). Participants were excluded from the analysis if they refused to pedal for the entire duration of the test.

#### Physical Activity and Sedentary Time

Time spent in LPA, MVPA, and in sedentary time was assessed as average minutes per day using an accelerometer (Actigraph GT3X+ activity monitor) worn around the waist. The devices were worn for seven consecutive days, except during sleep or prolonged water-based activities (e.g., bathing, swimming). The parents were asked to keep a record of the time of day that the monitor was placed on and removed each day. Data were analysed using ActiLife 6 software (ActiGraph, Pensacola, FL, United States) and have been previously described (25). Due to the short sporadic activity bursts typical of young children, 3-s epochs were used. Children who did not meet the accelerometer wear time criteria (≥10 h per day for 3 or more days per week) were removed from the analysis. Sedentary time as well as the time spent in LPA and MVPA were determined using the 2008 Evenson activity cut points for children. These established cut points have been validated in the children aged 5–8 (34) and they have been shown to have the highest accuracy in activity classification in children and adolescents aged 5–15 (35).

#### Demographic and Health-Related Questionnaires

The primary parents/guardians of the enrolled children were asked to complete questionnaires regarding family demographics, level of education of themselves and their partners, and whether children had a diagnosis of one or more of the following: asthma or reactive airway disease, speech or language issues, non-corrected hearing issues, ear tubes or non-corrected vision issues.

#### Identifying Children at Risk for Developmental Coordination Disorder

The MABC-2 was used to assess motor coordination and to identity children with rDCD based on the established threshold cut points (≤16th percentile) (36). The MABC-2 is a standardised and validated test for the children and the adolescents aged 3–16 years (37). The test is individually administered including eight motor tasks across the following three categories: manual dexterity (three tasks), aiming and catching (two tasks), and balance (one static and two dynamic). The raw scores on these items are converted into standardised scores based on the child’s chronological age, and converted into an overall percentile. Children who score at or below the 16th percentile are categorised as rDCD and those scoring above the 16th percentile are categorised as TD. The test–retest reliability and standard of error of measurement for the standardised test scores have been reported as 0.80 and 1.30 (corresponding to 0.45 SDs), respectively (38).

### Statistical Analysis

All descriptive data are presented as mean ± SDs and proportions. The data were verified for normality through the Shapiro–Wilk and Kolmogorov–Smirnov tests and visual inspections of histograms and residuals Q–Q plots. Independent *t*-tests were used to compare baseline data between the analytical sample and those with missing data from the full cohort. Bivariate Pearson’s correlations were conducted between body adiposity [total body fat percentage (%)] and demographic variables, aerobic and anaerobic fitness, MABC-2, physical activity levels, and sedentary behaviour. The strength of correlation coefficients was interpreted as ≤0.10 small association; ≤0.30 moderate association; >0.50 large association. The independent variables that showed an association (*p* < 0.20) with the dependent (body adiposity) in the bivariate analysis, and that are not mediators in the causal pathway between health-related fitness and body adiposity, were considered confounding variables and included in regression analyses as covariates. We fitted general linear regression models to examine crude (unadjusted) and adjusted associations between aerobic and anaerobic fitness, physical activity and body adiposity. The assumption for linear regressions were verified. In the cases where standardised residuals ±3 SD, data were included if the values were physiologically plausible. There were no significant interactions by sex or MABC-2 total percentile score and body adiposity, aerobic fitness or physical activity levels, and therefore all primary analyses were conducted in the full analytical cohort. Linear regression models were used to assess the associations between aerobic and anaerobic fitness, physical activity and sedentary time and body adiposity (Model 1), and after adjusting for confounding variables. Age and sex were entered as covariates in Model 2 and age, sex, and MABC-2 total percentile score were entered as covariates in Model 3. To aid interpretation of the analyses, the results are expressed as predictive margins for body adiposity for increments in (a) aerobic fitness (seconds on Bruce treadmill test), (b) anaerobic fitness (W/kg from mean 30 s power on Wingate test), (c) sedentary time (min/day), and (d) MVPA (min/day). The coefficients for predicative margins were generated using all participant data. To limit estimates to realistic values of body adiposity, predictive margins estimates were restricted to body adiposity values between the 5th and 95th percentiles of the body fat percentage distribution. While there was no interaction between MABC-2 and body fatness, we stratified the predictive models by MABC-2 classification for illustrative purposes. Data were analysed using Statistical Package for the Social Sciences (SPSS version 25.0; IMB Corp., Armonk, NY, United States). The predictive margins were performed using Stata 16.1; *p*-values were based on two-sided tests and considered statistically significant at *p* < 0.05.

## Results

### Participant Characteristics

The analytical sample included 495 participants (83% of the enrolled cohort). Overall, sociodemographic characteristics, health-related fitness variables, physical activity, and sedentary behaviour variables for the enrolled cohort and the analytical sample are presented in Table 1. The majority of children had no medical conditions at baseline with asthma or reactive airway disease in 50 children (10%) and speech or language issues in 34 children (7%). The number and nature of diagnoses did not influence any outcome. Children with and without valid data for all included outcomes did not differ in sociodemographic characteristics; however, the analytical sample had statistically significantly higher scores for the overall MABC-2 total test percentage and each component of the MABC-2. These differences were not considered clinically meaningful. The reasons for missing data included: refusal or unable to undertake BIA (*n* = 5), did not achieve a maximal heart rate >180 bpm (*n* = 4), refusal or unable to pedal >25 rpm on the Wingate test (*n* = 11), equipment failure/data error on Wingate (*n* = 6), did not meet minimum wear criteria for the accelerometer (*n* = 75). On average, the analytical sample were 5.0 ± 0.6 years, 56% male, had a mean body adiposity of 22.7 ± 4.2% and mean aerobic fitness (time-to-exhaustion on the Bruce treadmill test) of 602.9 ± 99.4 s (Table 1). This level of aerobic fitness falls within the 50th–75th and 25th–50th percentile for boys and girls aged 5 years, respectively (39). Further description of the full CATCH baseline cohort has been previously published (27).

**TABLE 1:** Cohort characteristics: demographics, health-related fitness, and physical activity.

	Enrolled	Analytical
	cohort	sample
	(*n* = 589)	(*n* = 495)
Age (years)	4.9 (0.6)	5.0 (0.6)
Sex [boy, *n* (%)]	338 (57%)	279 (56%)
BMI percentile	56.1 (27.1)	55.8 (27.5)
**Parental education:**		
Bachelor’s degree or higher [*n* (%)]	365 (62%)	318 (64%)
Bachelor’s degree or higher, partner [*n* (%)]	274 (47%)	242 (49%)
MABC-2 total test percentile score	33.2 (29.1; *n* = 588)	35.4 (29.5)*
Manual dexterity	41.4 (29.1; *n* = 588)	43.2 (29.5)*
Aiming and catching	38.1 (26.6)	40.2 (26.6)*
Balance	33.4 (30.1; *n* = 588)	35.1 (30.4)*
Grouping, rDCD [*n* (%)]	288 (49%)	223 (45%)*
Body adiposity (%)	22.8 (4.2; *n* = 583)	22.7 (4.2)
**Aerobic fitness**		
Total treadmill time (s)	596.5 (102.2; *n* = 585)	602.9 (99.4)*
**Anaerobic fitness**		
Mean 30 s normalised power (W/kg)	3.3 (1.2; *n* = 572)	3.3 (1.2)
**Physical activity and sedentary behaviour (min/day)**		
Average sedentary time	451.7 (46.1; *n* = 514)	451.5 (45.7)
Average light physical activity time	200.6 (28.3; *n* = 514)	200.6 (28.2)
Average moderate-vigorous activity time	71.7 (19.8; *n* = 514)	71.7 (19.8)

Data are means (SD; n) *p < 0.05 independent t-test (continuous data) or Chi-squared (categorical data) between analytical sample and sample not included due to missing data.

### Bivariate Correlations and Simple Regressions

The bivariate associations between body adiposity, demographic variables, health-related physical fitness variables, MABC-2 test scores, physical activity and sedentary behaviour are presented in Supplementary Table 1. There was a strong association (*r* = 0.647, *p* < 0.001) between body adiposity and sex, with girls having ∼5% higher body fat than boys. There was no association between BMI percentile and body adiposity. There was a small negative association between body adiposity and age (*r* = −0.187, *p* < 0.001), aerobic fitness (*r* = −0.285, *p* < 0.001), and anaerobic fitness (*r* = −0.217, *p* < 0.001). A moderate inverse association was observed between body adiposity and MVPA (*r* = −0.344, *p* < 0.001). Small positive associations were found between body adiposity and mean daily sedentary minutes (*r* = 0.184, *p* < 0.001) and LPA (*r* = 0.111, *p* = 0.012). There were no associations between body adiposity and total MABC-2 test percentile score. Table 2 shows the crude (unadjusted) associations between body adiposity, demographics, aerobic and anaerobic fitness, MABC-2 test scores, physical activity and sedentary time, stratified by sex. In both boys and girls, age (β = −1.058 for boys; β = −1.606 for girls, *p* < 0.001), MABC-2 total test percentile score (β = −0.023 for boys, *p* < 0.001; β = −0.015 for girls, *p* = 0.032), aerobic fitness (β = −0.010 for boys; β = −0.011 for girls, *p* < 0.001), and anaerobic fitness (β = −0.612 for boys; β = −0.979 for girls, *p* < 0.001) were negatively associated with body adiposity. MVPA was associated with body adiposity in boys only (β = −0.040, *p* < 0.001).

**TABLE 2:** Crude associations of demographics, health-related physical fitness variables, motor coordination, physical activity, and sedentary time with body adiposity for boys and girls.

	Boys	*p*-Value	Girls	*p*-Value
	β (95% CI)		β (95% CI)	
Age (years)	−1.058 (−1.642, −0.473)	<0.001	−1.606 (−2.240, −0.972)	<0.001
BMI percentile	0.003 (−0.010, 0.016)	0.650	0.012 (−0.003, 0.027)	0.118
Number of diagnoses at baseline	0.330 (−0.109, 0.769)	0.140	−0.284 (−1.024, 0.456)	0.450
MABC-2 total test % score	−0.023 (−0.035, −0.010)	<0.001	−0.015 (−0.028, −0.001)	0.032
Aerobic fitness (treadmill time, s)	−0.010 (−0.013, −0.007)	<0.001	−0.011 (−0.015, −0.007)	<0.001
Anaerobic fitness (W/kg)	−0.612 (−0.894, −0.329)	<0.001	−0.979 (−1.324, −0.635)	<0.001
Sedentary time (min/day)	0.009 (0.001, 0.017)	0.026	0.004 (−0.005, 0.014)	0.366
Light physical activity (min/day)	−0.002 (−0.016, 0.012)	0.768	0.008 (−0.007, 0.022)	0.310
Moderate-vigorous physical activity (min/day)	−0.040 (−0.058, −0.022)	<0.001	−0.023 (−0.048, 0.001)	0.061

*Crude unadjusted associations between body adiposity and independent variables. β = unstandardized beta. BMI, body mass index (kg/m^2^); MABC-2, movement assessment battery for children – Second Edition.*

### Multiple Regressions

**FIGURE 1:**
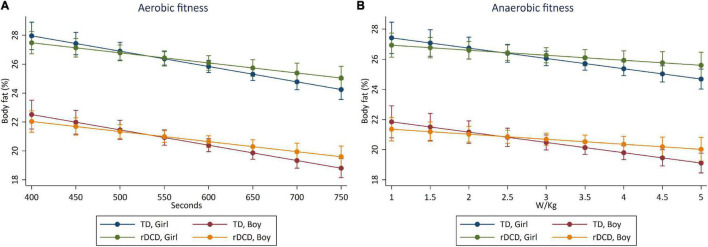
Predictive margins for reductions in body adiposity (%) with increments in **(A)** aerobic fitness (time on Bruce test, s) and **(B)** anaerobic fitness [Wingate mean 30 s power (W/kg)]. Cross-sectional association between aerobic fitness **(A)** and anaerobic fitness **(B)** with body adiposity in boys and girls classified as TD and rDCD. The highest values on the *x*-axes represent the 95th percentile of aerobic fitness **(A)** and anaerobic fitness **(B)** distributions.

**FIGURE 2:**
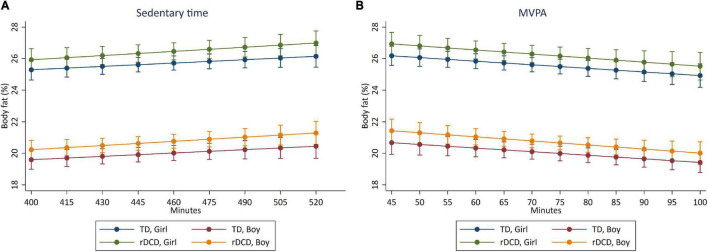
Predictive margins for reductions in body adiposity (%) with increments in **(A)** sedentary time (min/day) and **(B)** MVPA (min/day). Cross-sectional association between sedentary time **(A)** and MVPA **(B)** with body adiposity in boys and girls classified as TD and rDCD. The highest values on the *x*-axes represent the 95th percentile of sedentary **(A)** and MVPA **(B)** distribution.

The crude and adjusted cross-sectional associations between aerobic and anaerobic fitness, sedentary time, LPA and MVPA and body adiposity are presented in Table 3 and Figures 1, 2. Aerobic fitness (β = −0.006, 95% CI: −0.009, −0.003, *p* < 0.001), and MVPA (β = −0.018, 95% CI: −0.036, <0.001, *p* = 0.045) were negatively associated with body adiposity, when adjusted for age, sex, and MABC-2 total test percentile score. Higher anaerobic fitness was associated with lower levels of body adiposity when adjusted for age and sex, but the association did not persist when adjusted by MABC-2 total test percentile score. The inverse cross-sectional associations of aerobic fitness (Figure 1A), anaerobic fitness (Figure 1B), and MVPA (Figure 2B) with body adiposity were consistent for boys and girls, for both TD and rDCD classifications. Similarly, the positive association between sedentary time and body adiposity was consistent across sex and developmental classification (Figure 2A).

**TABLE 3:** Associations between aerobic and anaerobic fitness, physical activity, and body adiposity.

	Model 1 β (95% CI)	*p*-Value	Model 2 β (95% CI)	*p*-Value	Model 3 β (95% CI)	*p*-Value
Aerobic fitness (treadmill time, s)	−0.007 (−0.011, −0.004)**	<0.001	−0.006 (−0.009, −0.003)**	<0.001	−0.006 (−0.009, −0.003)**	<0.001
Anaerobic fitness (W/kg)	−0.199 (−0.529, 0.132)	0.238	−0.300 (−0.569, −0.030)*	0.029	−0.248 (−0.535, 0.038)	0.089
Sedentary time (min/day)	0.003 (−0.007, 0.012)	0.569	0.006 (−0.002, 0.013)	0.132	0.006 (−0.002, 0.013)	0.124
Light physical activity (min/day)	0.006 (−0.009, 0.020)	0.448	0.011 (<0.001, 0.022)	0.060	0.010 (−0.001, 0.021)	0.066
Moderate-vigorous physical activity (min/day)	−0.063 (−0.086, −0.040)**	<0.001	−0.018 (−0.036, 0.1 × 10^–2^)*	0.045	−0.018 (−0.036, 0.1 × 10^–2^)*	0.045
*R* ^2^	0.160**	<0.001	0.523**	<0.001	0.524**	<0.001
Adjusted *R*^2^	0.152**	<0.001	0.516**	<0.001	0.516**	<0.001

**p < 0.001, *p < 0.05. β = unstandardised beta. Model 1: crude associations; Model 2: adjusted for age and sex; Model 3: adjusted for age, sex, and MABC-2 total test percentile score.

## Discussion

The aim of this study was to examine the cross-sectional associations among aerobic and anaerobic fitness, device-measured physical activity and sedentary time, and body adiposity using laboratory-based measures in early childhood. We further explored these associations in children classified as TD and at rDCD. Across our whole sample that included 495 children (83% of enrolled cohort, 56% boys), we observed that aerobic fitness and MVPA were inversely associated with body adiposity and that these relationships were consistent across sex and motor coordination classification. Higher anaerobic fitness was also associated with lower body adiposity, but the association did not persist when adjusted by MABC-2 score in our final model. LPA showed no association. Contrary to our hypothesis, we did not observe a relationship between mean daily time spent in sedentary time and body adiposity. Consistent with previous findings (20), the BMI percentile had no association with the body adiposity in this population.

This is the first study to investigate these relationships in an early childhood cohort, and our observations highlight that the relationships between physical fitness, MVPA, and body adiposity are present even in early life stages, regardless of motor coordination. Increasing aerobic fitness and levels of MVPA are therefore important dual targets for lifestyle recommendations in all children of preschool age. These data reinforce the observations in older childhood and adolescents that physical fitness is a potent marker of health in children and adolescents (10). In consonance, Henriksson et al. (21) demonstrated that a higher fat mass index (fat mass (kg)/height^2^ (m), *via* air displacement plethysmography) was associated with lower cardiorespiratory fitness (*via* 20 m shuttle run; β = −0.17, *p* = 0.002), lower body muscle strength (through standing long jump; β = −0.17, *p* = 0.003), and lower motor fitness (*via* 4 m × 10 m shuttle run; β = −0.21, *p* < 0.001), when adjusted for age, sex, and both the fat- and fat-free mass indices. Cardiorespiratory fitness was also beneficially associated with the fat-free mass index. Collectively, these data demonstrate that lean body mass and body adiposity have opposing associations with health-related components of physical fitness.

School-aged children with DCD have increased levels of obesity (23), lower cardiorespiratory fitness, with a higher likelihood of being below the 20th percentile for V̇O_2peak_ (40), and lower physical activity levels (41, 42) than TD peers. Our findings demonstrated that the inverse relationship between aerobic fitness and body adiposity was not different between children at risk of DCD and TD children at this young age. This is consistent with observations, using the same CATCH cohort sample by King-Dowling et al., that preschool-aged children at risk for DCD are no less active than their TD peers (25), potentially due to the relative low skill requirement for motor behaviour involved in play and activity at this age. Importantly, children with DCD will be increasingly challenged by the higher motor skill demands of activity and play (43). Whether early intervention targetting physical fitness and appropriate levels of MVPA in rDCD children can prevent the observed decline in physical activity and fitness and increased levels of overweight/obesity is yet to be elucidated and requires longitudinal investigation.

Our findings should be considered in the context of study limitations. While time-to-exhaustion on the Bruce maximal graded exercise test is strongly correlated with direct measurement of V̇O_2 max_ using indirect calorimetry (44), the young age of the cohort prohibited us from obtaining a gold-standard V̇O_2peak_ result. To verify the maximal nature of the tests, we excluded children who did not reach maximal hearts rates 180 bpm or more (33). Notably, the time on test outcome for TD children were consistent with published reference data in preschool children (39). Body adiposity was determined *via* bioelectric impedance analysis, based on differences in tissue resistance to an electrical current when conducted through lean or fat tissue. Total fat mass is calculated by subtracting fat-free mass from total body mass and lean mass measures are influenced by hydration status. Hydration status was verbally confirmed and if required, the bladder voided, to minimise the influence of hyper-hydration. Due to the nature of the larger cohort study, children were tested at various time throughout the day, and as the testing protocols involved physical fitness measures, fasting conditions were not appropriate. Moreover, given that fat-free mass and body adiposity were both derived from the BIA method, we were unable to examine independent associations with indices of fat-free mass. Further, we did not examine the association of physical activity as the independent variable with body fatness and physical fitness as dependents; future studies could examine these associations to understand the comparable effects of physical activity on fatness and fitness in children, regardless of their motor coordination capabilities. As is common in cohort studies, our analytical sample had significantly higher aerobic fitness and MABC-2 percentile scores; however, the mean differences were not clinically meaningful. As detailed in Cairney et al. (28), the relatively homogenous sample consists of predominantly white and English-speaking children, who had higher average levels of family education and a relatively low proportion of families with low income. By the design of the prospective cohort study, the children at risk for DCD were oversampled to achieve an approximately equal distribution of children classified with typical motor coordination and atypical motor coordination, which would not be expected in a contemporary cohort of children (expected prevalence 16% based on the MABC-2 threshold). Moreover, given the time course of diagnosis of DCD, which requires repeat motor assessment, not all those classified as rDCD would meet the DCD criteria in future years. Finally, residual confounding variables due to unmeasured outcomes cannot be ruled out. For example, genetics, socio–environmental factors, energy intake and dietary composition may also independently influence body adiposity.

From a public health perspective, a key implication of these findings is that early intervention is vital for pre-school aged children who have poor physical fitness and/or physical activity deficits, regardless of their motor coordination abilities, who have poor physical fitness and/or physical activity deficits. Newly promoted 24 h movement guidelines for children and young people (aged 5–17 year) encourage several hours of LPAs and limiting sedentary time to no more than 2 h/day in addition to promoting 60 min of MVPA (45). The present study highlights that for body adiposity, implementing lifestyle strategies early in life that specifically target activates to improve aerobic fitness and promote MVPA (rather than LPA or sedentary time) may potentiate life-long cardioprotective habits. This would likely also positively influence other variables of body composition such as bone mineral content and density and lean mass in young children. Further highlighting the importance of an early-age approach, Baquet et al. observed that adolescents who were less active at baseline, but increased physical activity at 4-year follow up, still did not reach the fitness levels of children who were more active at baseline (46).

In adults, there is clear and consistent evidence for the relative importance of aerobic fitness, arguably one of the most important single health outcomes across the lifespan, for cardiovascular events and all-cause mortality (47). Accordingly, the American Heart Association have called for cardiorespiratory fitness to be assessed as a “vital sign” in clinical practice (48). As levels of cardiorespiratory fitness in youth are strongly and inversely associated with future cardiometabolic risk profiles (49), considering cardiorespiratory fitness as a vital sign in children is also warranted. Currently, public health guidelines and strategies for children focus on accruing at least 60 min of MVPA daily; however, there are no specific recommendations regarding cardiorespiratory fitness. Standardising measurement and promoting age and sex-specific criteria based on normative data could be a future addition to guideline materials. In early childhood, an increased adoption of valid and reliable physical fitness assessment tools that are feasible to conduct outside of the laboratory setting, such as the PREFIT battery (50), could help identify children requiring targetted intervention and clinical follow-up.

Beyond achieving MVPA targets, activities that promote improvements in aerobic fitness should be advocated in early childhood. However, activities that increase aerobic fitness in adults (e.g., moderate intensity continuous training and/or structured high intensity interval training) do not align with the unstructured nature of child’s play, making recommendations challenging. In early childhood, encouraging risky independent play may provide children with a greater opportunity for more vigorous-intensity activity, while being more fun.

## Conclusion

In preschool-aged children, lower levels of aerobic fitness and MVPA were associated with higher levels of body adiposity, regardless of motor coordination classification. Early-life interventions promoting current MVPA targets and improvements in aerobic fitness are therefore warranted in both typically and atypically developing pre-schoolers to prevent obesity-related morbidity and mortality. A longitudinal follow-up is required to examine whether improving and/or maintaining a high aerobic fitness in children with DCD prevents the decline in physical activity and physical fitness and associated levels of overweight and obesity typically observed in this population.

## Data Availability Statement

The datasets presented in this article are not readily available. Where applicable, anonymized data might be provided. Requests to access the datasets should be directed to SK, s.keating@uq.edu.au.

## Ethics Statement

The study was approved by the Hamilton Integrated Research Ethics Board. Written informed consent to participate in this study was provided by the participants’ legal guardian/next of kin.

## Author Contributions

SK, GM, and JC conceived and designed the study, conducted the analyses and wrote the first draft of the manuscript, contributed to data interpretation, and approved the final manuscript. SK-D, BT, and MK contributed to survey design, data collection and interpretation, and reviewed, edited, and approved the final manuscript. All authors contributed to the article and approved the submitted version.

## Conflict of Interest

The authors declare that the research was conducted in the absence of any commercial or financial relationships that could be construed as a potential conflict of interest.

## Publisher’s Note

All claims expressed in this article are solely those of the authors and do not necessarily represent those of their affiliated organizations, or those of the publisher, the editors and the reviewers. Any product that may be evaluated in this article, or claim that may be made by its manufacturer, is not guaranteed or endorsed by the publisher.
