# Supplementary material for: Associations Between Fitness, Physical Activity, and Fatness in Preschool Children With Typical and Atypical Motor Coordination
Source: Front Pediatr. 2022 Apr 15;10:756862. doi: 10.3389/fped.2022.756862 (PMC9051235; doi:10.3389/fped.2022.756862)
Supplement: Supplementary file 1 [file Table_1.DOCX]

**Appendices 1: Supplementary Table 1. Relationships between body adiposity, demographics, health-related physical fitness variables, MABC test percentile scores and physical activity and sedentary time.**

|  | 1 | 2 | 3 | 4 | 5 | 6 | 7 | 8 | 9 | 10 |
| --- | --- | --- | --- | --- | --- | --- | --- | --- | --- | --- |
| 1. Body adiposity (%) |  |  |  |  |  |  |  |  |  |  |
| 2. Waist (cm) | .332** |  |  |  |  |  |  |  |  |  |
| 3. Age (yrs) | -.187** | .303** |  |  |  |  |  |  |  |  |
| 4. Sex | -.647** | .129** | .004 |  |  |  |  |  |  |  |
| 5. Aerobic Fitness (s) | -.285** | .018 | .400** | .047 |  |  |  |  |  |  |
| 6. MABC-2 total test (percentile score) | -.009 | -.073 | .198** | -.186** | .299** |  |  |  |  |  |
| 7. Anaerobic Fitness (W/kg) | -.217** | .143* | .508** | .006 | .442** | .422** |  |  |  |  |
| 8. Av min sed/day | .184** | -.077 | .066 | -.169** | -.097* | .056 | .162** |  |  |  |
| 9. Av min light/day | -.111* | .185** | -.023 | .197** | .052 | -.094* | -.151** | -.042 |  |  |
| 10. Av min MVPA/day | -.344** | .101* | .173* | .310** | .204** | -.006 | -.118** | .099* | .056 |  |

** p<0.01, * p<0.05 Sample size per variable are numbers indicated for full cohort in Table 1.
